# The Collateral Effects of COVID-19 Pandemic on the Status of Carbapenemase-Producing Pathogens

**DOI:** 10.3389/fcimb.2022.823626

**Published:** 2022-03-17

**Authors:** Carole Ayoub Moubareck, Dalal Hammoudi Halat

**Affiliations:** ^1^ College of Natural and Health Sciences, Zayed University, Dubai, United Arab Emirates; ^2^ Department of Pharmaceutical Sciences, School of Pharmacy, Lebanese International University, Bekaa, Lebanon

**Keywords:** carbapenemases, antimicrobial resistance, COVID-19, NDM, KPC

## Abstract

The serious challenge of antimicrobial resistance continues to threaten public health and lingers in the era of the coronavirus disease 2019 (COVID-19), declared pandemic by the World Health Organization. While the pandemic has triggered the importance of infection control practices and preventive measures such as physical distancing, hand hygiene, travel reduction and quarantine, the ongoing alarm of antimicrobial resistance seems to accompany the pandemic too. Antimicrobial resistance has been fostered during COVID-19, possibly due to high rate of empirical antibiotic utilization in COVID-19 patients, increased use of biocides, and the disruption of proper healthcare for other conditions. Specifically, carbapenemase-producing Gram-negative bacteria have shown to cause secondary bacterial infections in patients hospitalized for COVID-19. Clinical and microbiological evidence of such infections is accumulating in different parts of the world. With the resilient nature of carbapenemases, their association with mortality, and the limited treatment options available, concerns regarding this group of antibiotic-hydrolyzing enzymes during the pandemic are expected to upsurge. While the additional burden carbapenemases exert on healthcare is worrisome, it remains hidden or abandoned among the various health consequences of the pandemic. The purpose of this minireview is to shed a light on carbapenemase-associated infections during such unprecedented time of COVID-19. A focused insight shall be made into carbapenemases, their implications for COVID-19 patients, and the features and consequences of co-infection, with a review of available evidence from pertinent literature. The importance of increased surveillance for carbapenemase-producers and optimizing their management in relation to the pandemic, shall be addressed as well.

## Introduction to Carbapenem Resistance and Implications in COVID-19

In its ranking of bacterial pathogens into critical, high, and medium priority, the World Health Organization (WHO) classifies carbapenem-resistant *Acinetobacter baumannii* and *Pseudomonas aeruginosa*, and carbapenem-resistant and third-generation cephalosporin-resistant *Enterobacteriaceae*, as critical priority bacteria (Tacconelli et al., 2018). Such definition reflects urgent need to develop new antibiotics against these pathogens, and indicates their multidrug resistant nature, making them a particular menace in hospitals, nursing homes, and among critically-ill patients (WHO, 2017). As such, high-resistance rates to carbapenems in Gram-negative pathogens inescapably complicate infections, and are propagated across different countries by mobile genetic elements (Brink, 2019). Hence, carbapenems, last resort antibiotics for complicated bacterial infections, are now threatened by widespread resistance (Potter et al., 2016). A primary mechanism of carbapenem resistance in gram-negative bacteria is expression of acquired carbapenemases, enzymes that hydrolyze these antibiotics. Carbapenemase production is especially challenging when encountered in members of *Enterobacteriaceae* family, which can rapidly spread and colonize patients in healthcare environments, and are often resistant to multiple antibiotic classes, resulting in restricted treatment options (Bonomo et al., 2018).

Parallel to the ongoing health overload of antimicrobial resistance in general and carbapenemases in particular, the world witnessed, in 2020, the unprecedented emergency caused by a novel coronavirus, that first appeared in Wuhan, China (Wang et al., 2020). This virus was identified as the severe acute respiratory syndrome coronavirus 2 (SARS-CoV-2), the etiological agent of the coronavirus disease 2019 (COVID-19) (Wu et al., 2020), which became domestically and internationally spread, and shortly declared pandemic by the WHO with additional impact on society, environment, economy, politics and global biosafety (Barbuddhe et al., 2020; Benach, 2021). Essentially an infectious disease, many other ailments remained hidden in shadows of COVID-19, namely pediatric health (Zemrani et al., 2021), geriatric health (Dhama et al., 2020), and mental well-being (Gambin et al., 2021; Magson et al., 2021). The pandemic has caused a remarkable disruption in healthcare and emergency services, drug shortages, and a surge in misinformation (Rusic et al., 2021), described as infodemic (Orso et al., 2020).

Antimicrobial resistance continues to flourish as another adversity during the pandemic, even though certain measures that prevent and control infections are accompanying COVID-19, and may cause decreased antimicrobial resistance (Rusic et al., 2021). These measures include enhanced hand hygiene, reduced travel, decreased incidence of infections due to social distancing, disinfection and protective measures, introduction of novel biomarkers (i.e., procalcitonin), decreased antimicrobial consumption due to fewer patient consultations, and reduction of critically ill patient transfer among countries (Donà et al., 2020; Monnet and Harbarth, 2020; Rawson et al., 2020; Martin et al., 2021). However, the pandemic is exacerbating antimicrobial resistance through increased use of environmental biocidal agents, potential risk of co-infection, unlicensed use of antibiotics, increased rate of empirical antimicrobial treatment for respiratory illness, decreased resistance surveillance due to diagnosis focus on COVID-19, and overloading of healthcare systems. Unfortunately, the weight of these factors on antimicrobial resistance appears to be major, and their negative impact should be monitored (Cantón et al., 2020).

The exponential growth in antibiotic use during COVID-19 is possibly inducing further pressure, contributing to the selection of antibiotic-resistant bacteria, including carbapenemase producers (Sellera et al., 2021). For example, in 2020, 68.9% of COVID-19 patients reported use of antibiotics prior to hospital admission, with a self-medication rate of 33.0% (Zavala-Flores and Salcedo-Matienzo, 2020). Evidence exists that COVID-19 patients experience co-infection with carbapenemase producers (Arcari et al., 2021), and that the pandemic impacted control and surveillance programs previously established against these organisms (Belvisi et al., 2021). As such, meticulous investigation of carbapenemase producers in COVID-19 patients and analysis of pandemic implications on these resilient pathogens are needed.

## Concise Review of Carbapenemases

The global dissemination of carbapenemases continues at alarming rates, threatening the once effective carbapenems. The most prevalent types KPC, VIM, IMP, NDM, and OXA-48 are routinely reported in many infections worldwide (Hansen, 2021). Historically, the first isolated carbapenemases early in the 1990s were the chromosomal imipenem-hydrolyzing enzyme (Yang et al., 1990) and NmcA (Nordmann et al., 1993) in *Serratia marcescens* and *Enterobacter cloacae* respectively. Later, plasmid-encoded carbapenemases emerged, and an epidemic involving KPC, MBLs, and OXA-48 carbapenemases occurred at the beginning of the 21^st^ century (Bush, 2018). Carbapenemases are classified within classes A, B and D of the Ambler structural classification (Suay-García and Pérez-Gracia, 2019), although some plasmid-encoded class C enzymes (AmpC variants) are linked to reduced imipenem sensitivity (Sawa et al., 2020). A concise presentation of the three major Ambler types of carbapenemases is shown below; further reading (Hammoudi Halat and Ayoub Moubareck, 2020) can be sought for a comprehensive review.

### Ambler Class A Carbapenemases

Presented initially by the chromosomal enzymes NmcA (Nordmann et al., 1993) and SME-1 (Naas et al., 1994), these are non-metallo β-lactamases with serine active moiety, and wide hydrolytic profile for all β-lactamases except cephamycins. They are inhibited by clavulanate, tazobactam and boronic acid derivatives (Hammoudi et al., 2014). The more important class A carbapenemases are the plasmid-encoded KPC and GES. KPC enzymes were initially described in *Klebsiella pneumoniae* but are now detected in various members of the *Enterobacteriaceae* family. The *bla*_KPC_ genes can be carried on mobile genetic elements such as transposons (e.g., Tn*4401b*) and plasmids (IncFII, IncL/M, and IncN). Often, organisms expressing KPC genes are simultaneously resistant to other antibiotics, such as quinolones and aminoglycosides, creating further challenges (Logan and Weinstein, 2017). Although over 20 different KPC variants are known, KPC-2 and KPC-3 persist as most common, and are linked to the major *K. pneumoniae* clone of sequence type (ST)-258 (Naas et al., 2016). The GES family of enzymes (Naas et al., 2016) includes many extended-spectrum β-lactamases (ESBLs), with few displaying carbapenemase activity and being prevalent, like GES-2 (Stewart et al., 2015), GES-4 (Yamasaki et al., 2017), GES-5 (Ayoub Moubareck et al., 2019), GES-6 (Botelho et al., 2015) and GES-20 (Garza-Ramos et al., 2015). The numerous point-mutant derivatives of GES-enzymes and their geographic spread indicate ongoing evolution and sheds darkness on antibiotic resistance, as these enzymes can become true carbapenemases by single point mutations.

### Ambler Class B Carbapenemases

These are metallo-β-lactamases (MBLs) that diverge from serine enzymes by their active site and catalytic features. MBLs require a bivalent metal ion like zinc for activity, and hydrolyze all β-lactams, including carbapenems, with the exception of aztreonam (Bahr et al., 2021). MBLs are not inhibited by clavulanic acid, tazobactam, or vaborbactam. The most well-known MBLs are the Verona integron–encoded MBL (VIM), *Pseudomonas* (IMP)-type and the New Delhi MBL (NDM). The spread of MBLs is attributed to their expression on mobile genetic elements such as integrons, plasmids and transposons (Hansen, 2021). The remarkable global spread of NDM remains a worrisome manifestation; initially isolated from a Swedish patient traveling to New Delhi (Yong et al., 2009), the prototype enzyme, NDM-1, became prominent in *K. pneumoniae* and *Escherichia coli*, then in *A. baumannii* and *P. aeruginosa* (Dortet et al., 2014). Currently, about 28 NDM variants have been described and spread despite efforts of containment (Farhat and Khan, 2020). Serious outbreaks from China, India, Pakistan and Bangladesh caused by NDM-producing *Enterobacteriaceae* are reported, and will cripple carbapenem utility (Usman Qamar et al., 2020).

### Ambler Class D Carbapenemases

Initially known as oxacillinases because of hydrolytic activity on oxacillin, methicillin and cloxacillin, the class D β-lactamases became a clinical concern. They possess variable but significant carbapenemase activity and are generally not inhibited by clavulanic acid, tazobactam, and sulbactam (Hansen, 2021). Currently, important OXA enzymes with carbapenemase activity belong to groups OXA-23-like, OXA-24/40-like, OXA-48-like, OXA-58-like, OXA-143-like and OXA-235 (Hammoudi Halat and Ayoub Moubareck, 2020). The first carbapenem-hydrolyzing oxacillinase, OXA-23, was plasmid-mediated and isolated from *A. baumannii* early upon clinical use of imipenem (Scaife et al., 1995). It is capable of hydrolysis of oxyiminocephalosporins, aminopenicillins, piperacillin, oxacillin, aztreonam and carbapenems (Kaitany et al., 2013). The locations of OXA-23 are both plasmid and chromosomal-based, usually flanked by the insertion sequence IS*Aba1*, which likely acts as strong promotor of gene expression (Turton et al., 2006). Aside to the *A. baumannii*-derived group of OXA carbapenemases, the OXA-48-type carbapenemases, first isolated in Turkey from *K. pneumoniae* (Poirel et al., 2004), are increasingly reported in enterobacterial species. These enzymes hydrolyze penicillins at a high level and carbapenems at a low level, sparing broad-spectrum cephalosporins, and are not susceptible to β-lactamase inhibitors (Poirel et al., 2012). OXA-48, OXA-181, OXA-232, OXA-204, OXA-162, and OXA-244, in this order, are the most common enzymes among the group. Genetically, OXA-48 is associated with different Tn*1999* variants on IncL plasmids; it is endemic in North Africa and the Middle East, while OXA-162 and OXA-244 predominate in Europe. OXA-181 and OXA-232 are associated with IS*Ecp1*, Tn*2013* on ColE2, and IncX3 plasmids, and are endemic in the Indian subcontinent and certain sub-Saharan African regions (Pitout et al., 2019).

## Carbapenemase-Producers Causing Co-Infections in COVID-19 Patients: A Review of Available Evidence

Previous experience shows that carbapenemase-producing bacteria can thrive in times of chaos (Livermore, 2021), as seen in countries with war, socioeconomic and immigrant challenges (Lafeuille et al., 2013; Rafei et al., 2014; Dandachi et al., 2019). In COVID-19 era, with all associated inconveniences, stressed healthcare systems, and collateral damage, an analogous portrait is expected. For example, a statistically significant difference in the resistance to carbapenems was noted in one study from Serbia (Despotovic et al., 2021) when COVID-19 patients were compared to non-COVID-19 patients in the prepandemic year. A significant association between the diagnosis of COVID-19 and a more exaggerated resistance to different carbapenems (imipenem, 56.8% *vs*. 24.5%, p < 0.001; meropenem, 61.1% *vs*. 24.3%, p < 0.001; and ertapenem, 26.1% *vs*. 21.7%, p = 0.03) was observed upon comparison. Such observation indicates the significant shift of resistance profiles with COVID-19, and may be attributed to the inability to completely conform with standard practices of infection control during such unprecedented time (Pascale et al., 2021). Also, the in-hospital use of antibiotics has known to be a driver of resistance in a time-dependent manner whether or not a pandemic takes place, highlighting the need for rational antimicrobial use across all levels of healthcare (Kousovista et al., 2021), and this indeed, applies to carbapenems. In one retrospective single-center, case-control study, authors from Spain reported that COVID-19 patients had increased risk of nosocomial infections with carbapenemase-producing *Enterobacteriaceae*, with infections being severe, appearing in critically-ill patients, and associated with a high mortality. The aforementioned infection was the cause of death in about two thirds of the studied patients (Pintado et al., 2022). In Brazil, the COVID-19 pandemic was correlated to an increase in incidence of carbapenem-resistant *A. baumannii* both in ICU and non-ICU settings (Polly et al., 2021). Likewise, the number of carbapenem-resistant *Enterobacteriaceae* isolated from COVID-19 patients in a Turkish hospital was higher than that isolated during an equal time period prior to the pandemic (Karataş et al., 2021). Taken together, such annotations indicate that institutions and regions with a high prevalence of carbapenemase-producers should be primed for a significant increase in the prevalence of these strains with the progressive expansion of the pandemic. Although similar carbapenemase groups are being isolated as in the pre-pandemic era, the implementation of increased surveillance and antimicrobial stewardship programs should continue throughout the pandemic. Additionally, with COVID-19, carbapenemase-producers are expected to renew their challenge (Livermore, 2021). Accordingly, and with subsequent pandemic waves and emergence of new SARS-CoV-2 variants, focused efforts to control carbapenemase-producers should be an essential component of management strategies for COVID-19 patients.

In an attempt to review the available literature on infections caused by carbapenemase-producers in COVID-19 patients, a PubMed search was conducted using the search terms carbapenemase, COVID-19, Gram-negative bacteria, *Enterobacteriaceae*, *Pseudomonas*, and *Acinetobacter*. All retrieved studies in 2020 and 2021 discussing infection of COVID-19 patients with carbapenemase-producers among these three bacterial categories were included, whether as co-infecting or secondary infecting bacteria. Positive results from samples collected within 2 days of admission were categorized as co-infections, and those collected more than 2 days after admission as secondary infections (Russell et al., 2021). Studies not mentioning carbapenemase production among co-infecting or secondary infecting bacteria, and studies in which COVID-19 patients were not distinguished for infection or colonization by carbapenemase-producers were excluded. According to this search, observation of bacterial infection with carbapenemase-producers in patients with COVID-19 started with the pandemic (Hughes et al., 2020; Ling et al., 2020; Nori et al., 2021), with detection of multiresistant Gram-negative pathogens (Chen et al., 2020) as well as carbapenemase producers (Cultrera et al., 2021; Sang et al., 2021; Senok et al., 2021). Early during COVID-19, a report from China (Zhang et al., 2020) found that more than half of COVID-19 patients in intensive care unit (ICU) of one hospital were coinfected with carbapenem-resistant *A. baumanni*, more common in patients who died compared to survivors. In a single-center experience from Italy, 50% critically ill COVID-19 patients developed multi-drug resistant infections, of which 32% were carbapenem-resistant *K. pneumoniae* (KPC-producing), 19% were carbapenem-resistant *A. baumannii*, and 14% were carbapenem-resistant *P. aeruginosa* (Karruli et al., 2021). With carbapenem-resistant bacteria considered critical priority pathogens, there is an urgency to revise available literature about carbapenemases in bacterial co-infections with COVID-19 and to refine findings for better surveillance and control. We present below a summary of studies on carbapenemase-producing *Enterobacteriaceae* and *A. baumannii* in COVID-19 patients. Regarding *P. aeruginosa*, and while this multiresistant pathogen is known to produce carbapenemases (VIM-2, GES-7, VEB-1, IMP-1, KPC-2, PER-1) (Cantón et al., 2020), and despite being among top pathogens co-infecting COVID-19 patients (Ramadan et al., 2020; Senok et al., 2021; Westblade et al., 2021), sometimes associated with severe pyogenic infection (Meguins et al., 2021), literature has not yet extensively reported carbapenemases from this organism in COVID-19 patients. This calls for vigilant investigations to fill such gap of knowledge regarding this significant nosocomial pathogen. In a small cohort of Italian COVID-19 patients on assisted ventilation, carbapenem-resistant *P. aeruginosa* in bronchial aspirates was the most common agent causing superinfection, and resistance was attributed to OprD porin mutation/deletion rather than production of carbapenemases (Mazzariol et al., 2021).

### Studies Involving *Enterobacteriaceae*

A summary of studies reporting co-infection of COVID-19 patients with carbapenemase-producing Gram-negative pathogens is shown in **Table 1**. Evidence obtained from studies on carbapenemase-producing *Enterobacteriaceae* is quite heterogeneous. Although some findings published early in the pandemic on COVID-19 patients didn’t detect significant rise in carbapenemase-producing *Enterobacteriaceae* compared to previous years (Cuntrò et al., 2021; Pascale et al., 2021), this depiction changed later with rise in these pathogens observed in many regions of the world. For example, colonization/infection with *Enterobacteriaceae* producing NDM in a cohort of COVID-19 patients in an Italian teaching hospital increased versus other patients, and co-infection significantly increased duration of hospital stay by about 17 days (Porretta et al., 2020). Also, incidence of acquisition of KPC-producing *K. pneumoniae* in COVID-19 patients from the ICU of another hospital increased from 6.7% in 2019 to 50% in 2020 (Tiri et al., 2020). It is possible that overloading of facilities, presence of many healthcare workers in a high risk area, extended and prolonged contact with patients, presence of untrained personnel, and increased use of empirical antimicrobial therapy contribute to such increase in colonization (Donà et al., 2020; Rawson et al., 2020; Tiri et al., 2020). In some instances, and in the setting of a recent decrease of carbapenemase-producing *Enterobacteriaceae* in the institution and the region, their rise in COVID-19 patients was notable. This supports concerns regarding emergence of carbapenemases in the wake of the global pandemic (Gomez-Simmonds et al., 2021). In other settings, a depicted change occurred in *K. pneumoniae* ST and its virulence traits over 5 years since 2015, where 2020 witnessed a dominant shift from ST11 to ST15, and this may be ascribed to COVID-19 (Chen et al., 2021). A review of studies on carbapenem-resistant *K. pneumoniae* in COVID-19 patients from 6 countries found that majority of infected patients (84%) were males with mean age of 61 years, and predominant carbapenemases were KPC and NDM. Cases described were from Italy (4 studies), China (2 studies), Egypt, United States, Spain, and Peru (one study from each). Different factors contributed to variable prevalence of carbapenemase-producers, ranging between 0.35-53%, with lowest prevalence reported in US and highest in China. It was hypothesized that contaminated protective equipment may be the main cause of cross-transmission, namely use of same protective facemasks to care for different patients, and use of double gloves where outer gloves were changed while inner gloves were disinfected with alcohol. Other factors included employment of additional medical staff unexperienced in infection control, use of broad-spectrum antibiotics in COVID-19 patients, and medical personnel burnout, decreasing commitment to infection prevention and relaxing rules of infection prophylaxis and surveillance (Mędrzycka-Dąbrowska et al., 2021).

**Table 1:** Summary of studies describing carbapenemase-producing Gram-negative pathogens reported in COVID-19 patients as of August 2021.

Species	Country	Sample type	Time of collection of study strains	Hospital ward	No. of co-infected patients/total (%)	Underlying patient diseases reported	Carbapenemase detected in COVID-19 patients; No. of patients/total	Mortality rate due to the specific pathogen	Other major findings	Reference
*Klebsiella pneumoniae*	Italy	Blood cultures and rectal swabs	March 6 to May 20, 2020	ICU	14/41 (34.14%)	Not specified	OXA-48; 10/14 KPC-3; 4/14	Not specified	Carbapenemase-producers were detected in 8% of blood cultures and 34.1% of rectal swabs.	(Arcari et al., 2021)
	Italy	Fecal material	February to April 2020	ICU	81% of ICU patients^a^	Not specified	KPC	Not specified	Prevalence of KPC-producing *K. pneumoniae* increased significantly in COVID-19 period (from Feb till April 2020) versus the previous decreasing trend, then normalized again (from April to June 2020).	(Belvisi et al., 2021)
	Italy	Pulmonary infiltrates	March 1 to May 20, 2020	ICU	7/35 (20%)	Obesity, hypertension, asthma, COPD, and hypothyroidism in 5 patients	Not specified	2/7 within 28 days	A mortality of 28.6% was due to carbapenemase-producing *K. pneumoniae* associated septic shock.	(Montrucchio et al., 2020)
	New York	Respiratory samples, blood, and urine	March 10 to April 30, 2020	ICU	10^b^/3152 (0.317%)	Chronic comorbidities	KPC-2; 16/18 KPC-3; 1/18 KPC of unavailable subtype; 1/18	4/10	94% of *K. pneumoniae* belonged to ST258 and were linked to historical isolates collected at the hospital prior to the pandemic.	(Gomez-Simmonds et al., 2021)
	Romania	Sputum, blood and urine	Not specified	ICU	9/25 (36%)	Hypertension (7 patients), hypothyroidism (3 patients), asthma obesity (2 patients) and others	OXA-48; 1/9 KPC; 4/9 OXA-48 and KPC: 4/9	5/9	KPC was never isolated in the hospital prior to the pandemic. Death correlated with age over 70 years, tocilizumab therapy, and the presence of carbapenemase-producing *K. pneumoniae* in sputum and blood	(Dumitru et al., 2021)
	France	Blood, respiratory samples, and rectal swabs	Mid-March to mid-May 2021	ICU	12 patients^c^	Not specified	NDM-1; 12/12	5/12	All isolates were clonally related and belonged to ST15, indicating an outbreak.	(Amarsy et al., 2021)
	Spain	Blood, respiratory material and rectal swabs	February to May 2020	ICU	7/62 (11.29%)	Hypertension, diabetes, ischemic heart disease, H1N1 influenza, asthma, epilepsy, and schizophrenia in 5 patients	OXA-48; 7/7	1/7	All strains belonged to ST236, a clone previously related to OXA-48 dissemination in Spain.	(García-Meniño et al., 2021)
	Peru	Endotracheal secretion	September 2020	ICU	4 patients^c^	Hypertension/hypothyroidism and obesity in two patients	NDM-1; 3/4	3/4	No previous cases with NDM were detected in the same hospital.	(Arteaga-Livias et al., 2021)
*Enterobacter cloacae*	New York	Respiratory samples	March 10 to April 30, 2020	ICU	3/3152 (0.095%)	Chronic comorbidities	NDM-1; 3/3	1/3	A single sequence type (ST270) with NDM-1 encoding gene carried by an IncHI2 plasmid.	(Gomez-Simmonds et al., 2021)
*Escherichia coli*	France	Rectal swabs	March to April 2020	ICU	6/25 (24%)	Not specified	NDM-5	Not specified	All isolates were clonally related and belonged to ST361, indicating an outbreak.	(Farfour et al., 2020)
*Acinetobacter baumannii*	Italy	Rectal swabs and respiratory samples	January to April 2020	ICU	55/1367 (4%)	Not specified	OXA-23	9/21	All strains belonged to the CC92/IC2 clonal lineage.	(Pascale et al., 2021)
	China	Respiratory samples, blood, and urine	January 27 to March 17, 2020	COVID-19 hospital	47/102 (46%)	Not specified	Not specified	Not specified	The isolation rate of carbapenem-resistant *A. baumannii* was 91.2%.	(Li et al., 2020)
	Egypt	Respiratory samples	May 3 to June 30, 2020	ICU and other units	7/260 (2.7%)	Hypertension, diabetes, kidney or liver disease, Parkinsonism, COPD or ischemic heart disease	NDM-1	Not specified	The resistance rates to imipenem and meropenem were 71% and 43% respectively	(Ramadan et al., 2020)
	Turkey	Respiratory samples and urine	March 15 to June 15, 2020,	Not specified	12/1447 (0.82%)	Not specified	Not specified	Not specified	*A. baumannii* was the main pathogen in respiratory infections of COVID-19 patients (9.76%)	(Karataş et al., 2021)
	Iran	Tracheal discharge and blood	March 30 to May 30, 2020	ICU	18/242 (7.4%)	Asthma, cardiovascular disease, lymphoma, hypothyroidism, diabetes, necrotizing fasciitis, hypertension or chronic kidney disease in 14 patients	OXA-23, 13/18 OXA-23 and OXA-24; 5/18	9/18	All isolates belonged to GC2 and were found to contain the same MLST profile of ST2_IP_.	(Abdollahi et al., 2021)
*Pseudomonas aeruginosa*	Egypt	Respiratory samples	May 3 to June 30, 2020	ICU and other units	4/260 (1.5%)	Hypertension, diabetes, kidney or liver disease, Parkinsonism, COPD or ischemic heart disease	NDM-1	Not specified	The resistance rates to both imipenem and meropenem were 100%.	(Ramadan et al., 2020)
	UAE	Blood, endotracheal aspirates, and others	February 1 to July 31, 2020	Not specified	48/29,802	Diabetes, hypertension, asthma, renal disease, neurologic disease, and others	Not specified	Not specified	*P. aeruginosa* formed the majority of carbapenem-resistant isolates (48/52) and the top organism isolated from positive cultures.	(Senok et al., 2021)

a Number of patients analyzed was not specified.

b Some patients had two or three strains of K. pneumoniae belonging to different ST.

c Total number of patients was not specified.

As such, international literature indicates that carbapenemase-producing *Enterobacteriaceae* may have lingered in the context of COVID-19, and focused studies may be needed to assess changes in their prevalence, molecular genetics, and dissemination. Also, rational antibiotic therapy should be used, as well as continuous assessment of co-infections in patients with COVID-19, to limit outbreaks with carbapenemase-producers that may ascend while managing the pandemic.

### Studies Involving *A. baumannii*

An agent of pneumonia, septicemia, meningitis, urinary tract and wound infections, carbapenemase-producing *A. baumannii* readily contaminates hospital facilities and healthcare personnel’s hands, survives on dry surfaces, and spreads from asymptomatic colonization. Collectively, these factors make its outbreaks challenging to control (Ayoub Moubareck and Hammoudi Halat, 2020). During surge in COVID-19 admissions in a hospital in New Jersey, 26 patients were co-infected with OXA-23-producing *A. baumannii*, 4 of which co-harbored NDM, rarely identified in *A. baumannii* from the US. As COVID-19 hospitalizations decreased, and standard infection control practices resumed, prevalence of carbapenem-resistant *A. baumannii* returned to pre-COVID-19 baseline of zero to two per month in New Jersey (Perez et al., 2020). Such shift indicates potential for carbapenemase producers to spread during events when typical hospital practices are disorganized, but limiting and decreasing spread are possible too. Similarly, an Italian multicenter before-after cross-sectional study that compared colonization and infection with carbapenem-resistant *A. baumannii*, detected increase of 7.5 and 5.5 folds respectively, with predominance of OXA-23 (Pascale et al., 2021). In Wuhan, China, among 159 strains of bacteria isolated form 102 COVID-19 patients with secondary bacterial infections, *A. baumannii* was the most common pathogen (35.8%) with carbapenem resistance rates above 90% (Li et al., 2020). In another COVID-19 report from Egypt, 100% of *A. baumannii* isolates harbored NDM-1, with 71% and 43% resistance rates to imipenem and meropenem respectively (Ramadan et al., 2020).

## Recommendations for Stewardship and Surveillance of Carbapenemase-Producers

Combating surge of carbapenemases during COVID-19, while conforming with general measures of antimicrobial stewardship, warrants additional alertness and investigation. Detection of carbapenemase producers in COVID-19 patients calls for infection control activities both intra- and inter-hospitals, and if necessary re-modulating them according to new organizational structures imposed by the pandemic (Pascale et al., 2021). In areas where carbapenemase producers have declined substantially in recent years, increased detection in patients with COVID-19 may signal a re-emergence, prompting need for increased surveillance and identification of optimal treatments (Gomez-Simmonds et al., 2021). The experience gained during the pandemic should be built upon to improve strict surveillance for carbapenemase producers colonization and co-infection in COVID-19 patients (Arcari et al., 2021). Consensus statements published recently by professional societies (Centers for Disease Control and Prevention (CDC), 2009; Tacconelli et al., 2019) and independent research groups (Chea et al., 2015; Puleston et al., 2020; Yarbrough et al., 2020) focused on detection of carbapenemase producers, which should be reinforced during COVID-19.

The optimal treatment of infections due to carbapenemase-producing organisms is uncertain, and options are quite limited (Rodríguez-Baño et al., 2018). In general, for infections caused by KPC- or OXA-48-producers, ceftazidime-avibactam (van Duin et al., 2018), or a novel beta-lactam-beta-lactamase inhibitor combination, such as meropenem-vaborbactam, imipenem-cilastain-relebactam (Zhanel et al., 2018), or cefiderocol (Zhanel et al., 2019) are suggested. Overall, clinical experience in treating carbapenemase-producing organisms with these agents is limited; most has been with ceftazidime-avibactam (Rodríguez-Baño et al., 2018). For MBL producers, and since these confer resistance to all beta-lactam-type antibiotics except cefiderocol and aztreonam, combining ceftazidime-avibactam and aztreonam can have a synergistic effect, as avibactam can inactivate other beta-lactamases to preserve aztreonam activity. This combination was used to successfully treat patients with extremely resistant MBL-producing pathogens (Marshall et al., 2017; Jayol et al., 2018). Alternative regimens include polymyxin with a second agent like meropenem (Paul et al., 2018), tigecycline or eravacylcine (Karakonstantis et al., 2020). Evidence indicates that these regimens were used in treatment of infections caused by carbapenemase-producers with variable outcomes (Montrucchio et al., 2020; Gomez-Simmonds et al., 2021). In one report, despite ceftazidime/avibactam therapy, five of nine patients infected with *K. pneumoniae* producing KPC and/or OXA-48 died, contributing to poor prognosis for patients with COVID-19, especially for high-risk populations (Dumitru et al., 2021). Further analysis is warranted to understand outcomes of treatment with these antibiotics in COVID-19 patients upon co-infection with carbapenemase-producers. It is crucial to establish a rigorous program of antibiotic administration and to maintain rational antibiotic use and continuous surveillance.

## Concluding Remarks

Carbapenemases contributing to antimicrobial resistance form a collateral and unavoidable consequence of COVID-19 and combating them remains demanding. In the aftermath of the pandemic, calls for global health security measures to limit spread of these enzymes will take on a new urgency, and the more powerful these measures are, the more delineated their outcomes will be on dissemination and thriving of carbapenemases. In pandemic emergencies, like the current one, antimicrobial stewardship shall contribute to better use of resources, limit unnecessary use of antimicrobials, and restrain antibiotic resistance trends including formidable challenges instigated by carbapenemases.

## Author Contributions

DHH and CAM designed this minireview. DHH wrote the first draft of the manuscript. CAM was responsible for editing, reviewing, and acquiring the funds. All authors contributed to the article and approved the submitted version.

## Conflict of Interest

The authors declare that the research was conducted in the absence of any commercial or financial relationships that could be construed as a potential conflict of interest.

## Publisher’s Note

All claims expressed in this article are solely those of the authors and do not necessarily represent those of their affiliated organizations, or those of the publisher, the editors and the reviewers. Any product that may be evaluated in this article, or claim that may be made by its manufacturer, is not guaranteed or endorsed by the publisher.
